# Benefits of using heated egg (HE) in the management of egg allergy

**DOI:** 10.1186/2045-7022-3-S3-O7

**Published:** 2013-07-25

**Authors:** A Claver, E Botey, B Navarro, D Nathalie, A Cistero-Bahima

**Affiliations:** 1 Allergy, Instituto Universitario Dexeus. UAB, Barcelona, Spain

## Background

Previous studies suggest that extensive heating and food matrix diminish the allergenicity of egg white proteins, making it possible to be tolerated by some children with egg allergy.

## Methods

50 patients (Table 1) underwent an OFC with HE performed in two different days. The first day began with gradual doses of cookies (a brand containing egg) and later home-breaded chicken. Subsequently, tolerant patients incorporated HE into their diet (cookies or food products containing egg daily and home-breaded foods 2-3 days/week). A second challenge with gradual doses of a serving size of a home-made cake containing 4 eggs was performed a week later. Regular consumption was advised for tolerant subjects. All children were periodically controlled. Factors including SPT and sIgE levels, psychological and others were used to determine a subsequent challenge with less-heated-egg (hard-boiled egg, omelette and raw consecutively).

**Table 1:** Baseline clinical characteristics

Gender	32 male/18 female
Mean age (y;range)	5.7 (0.5-16)
History of immediate reaction	50 (28 anaphylaxys, 13 cutaneous, 9 gastrointestinal)
Mean SPT to ovomucoid (OVM) and raw egg white (EW) (mm; range)	OVM: 7.39 (0-18.5) EW: 10.7 (5.5-18.5)
Mean OVM and EW sIgE levels (KU/L; range)	OVM: 8.86 (0.1-100) EW: 11.53 (0.1-100)

## Results

100% of patients tolerated cookies (2-4 units). 90% (45/50) tolerated breaded chicken in the first OFC; 2 patients presented mild anaphylaxis (1 adrenalin dose was required) and 3 mild abdominal pain treated with oral antihistamines. They were instructed a regular cookie intake and 3 were successfully re-challenged a month later. 91.6% (44/48) tolerated cake. 4 patients presented mild abdominal pain. In the next 6-12 months 72% (36/50) underwent an OFC with hard-boiled egg (negative in 29/5 gastrointestinal symptoms/1 cough/1 angioedema). 30% were challenged with omelette (14 passed/1 vomiting) and 12% with raw egg (all 6 passed). During the home dosing, 20% presented mild abdominal pain with excellent response to oral antihistaminic (50% of which later achieved hard-boiled egg tolerance). SPT wheal diameter to OVM and raw egg white and OVM sIgE levels decreased significantly from the baseline in patients tested. All patients, including the 2 that only tolerated cookies, reported a substantial improvement in quality of life.

## Conclusion

HE is well tolerated and safe. Thus, strict dietary avoidance may not be necessary at any stage of the protocol, including at the start.

## Disclosure of interest

None declared.

